# VISTA in hematological malignancies: a review of the literature

**DOI:** 10.3389/fimmu.2024.1466839

**Published:** 2024-12-17

**Authors:** Yuanjia Duan, Xiaotong Ren, Xinyu Guo, Jiayi Xie, Zhaoyun Liu, Lijuan Li

**Affiliations:** ^1^ Department of Hematology, Tianjin Medical University General Hospital, Tianjin, China; ^2^ Tianjin Key Laboratory of Bone Marrow Failure and Malignant Hemopoietic Clone Control, Tianjin, China; ^3^ Tianjin Institute of Hematology, Tianjin, China

**Keywords:** immune checkpoint receptors, VISTA, hematological malignancies, tumor microenvironment, immunotherapy

## Abstract

In recent years, tumor immunotherapy has become an active research area, with the emergence of immune checkpoint inhibitors (ICIs) revolutionizing immunotherapy. Clinical evidence indicates that programmed cell death protein 1 (PD-1) monoclonal antibodies and other drugs have remarkable therapeutic effects. V-domain Ig suppressor of T-cell activation (VISTA) is a new type of immune checkpoint receptor that is highly expressed in various tumors. It is co-expressed with PD-1, T-cell immunoglobulin domain, mucin domain-3 (Tim-3), T-cell immunoglobulin, and immunoreceptor tyrosine-based inhibitory motif domain (TIGIT) and is associated with prognosis, which suggests that it may be a target for immunotherapy. As an immune checkpoint receptor with no mature drugs, VISTA is highly expressed in acute myeloid leukemia (AML), multiple myeloma (MM), and other hematological malignancies; however, its pathogenic mechanism should be defined to better guide treatment.

## 1 Introduction

The pathogenesis of hematology-related tumors primarily includes genetic susceptibility, viral infection and immune system disorders, among which the immune system dysfunction has an important role in the etiology and development of hematology-related tumors. The immune system maintains the homeostasis of the body’s internal environment and guarantees normal physiological activities of cells and tissues, whereas immune cells promote the proliferation and invasion of tumor cells through complex mechanisms. Immune checkpoint receptors (ICRs) are a class of immunosuppressive molecules. A high ICR expression results in T-cell exhaustion, which reduces immunosurveillance and the killing of tumor cells, resulting in tumor immune escape (1). The V-domain Ig suppressor of T-cell activation (VISTA) is a type of immune checkpoint, of which mechanism of action in tumors has not yet been fully elucidated. In this review, the role of VISTA in hematological malignancies is summarized along with progress in hematological malignancies affecting the development of new treatment regimens.

## 2 Molecular biology of VISTA

### 2.1 Structure

**Figure 1:**
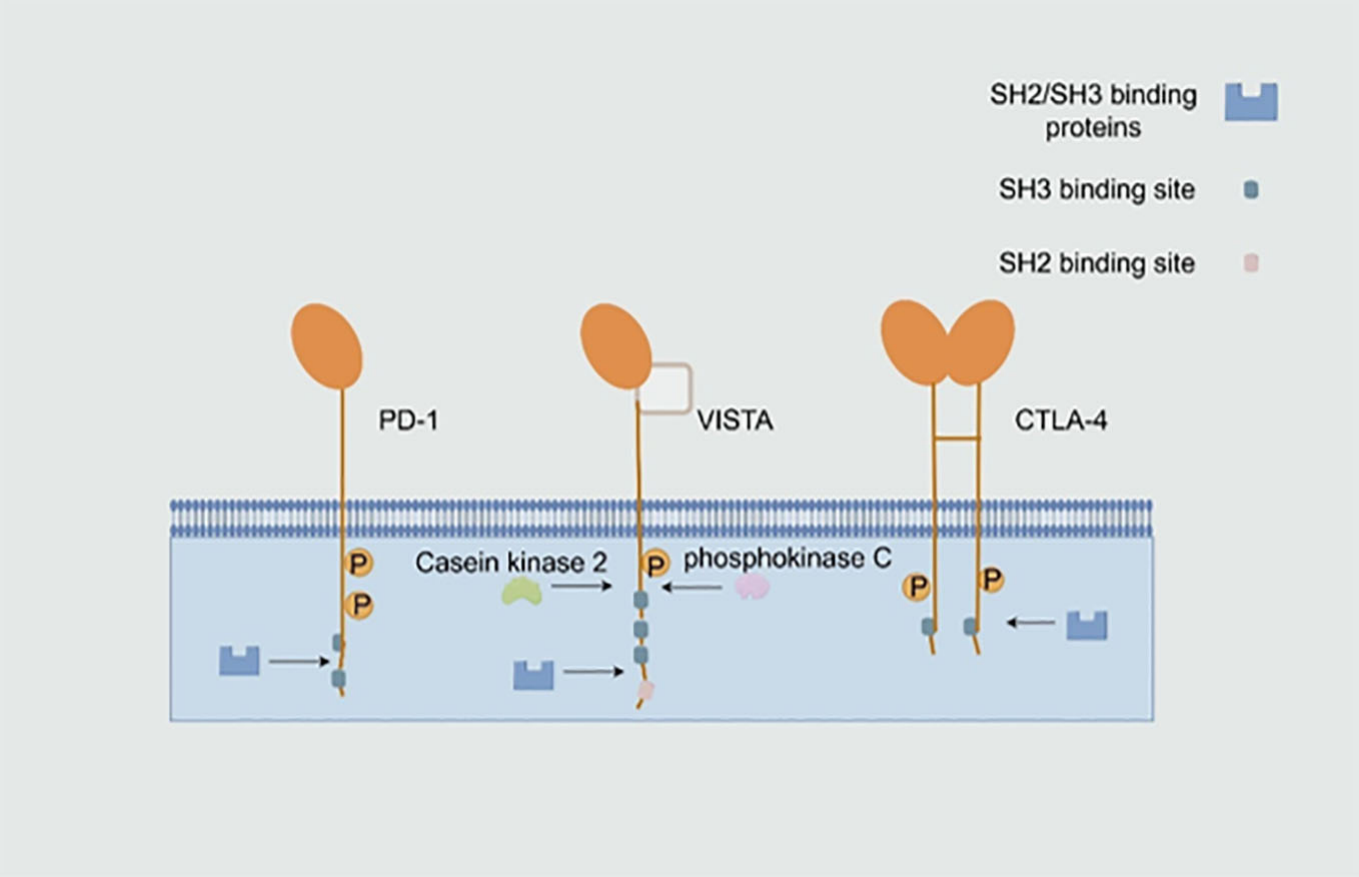
Structure of the V-domain Ig suppressor of T-cell activation (VISTA) and other immune checkpoint receptors. VISTA is a Type I transmembrane protein (3) that bears the features of both B7 and CD28 families of immunoregulatory molecules. Because of its single large IgV-like domain, VISTA has the highest homology with programmed cell death protein 1 (PD-1), a member of the CD28 superfamily. The intracellular tail of VISTA contains two potential protein kinase C-binding sites and a proline-rich motif, which may serve as a docking site (7). SH2, Src homology domain 2. (By Figdraw).

VISTA is also known as PD-1H, B7-H5, Dies1, Gi24, DD1α and C10orf54 and is encoded by the *VSIR* gene in humans and *Vsir* in mice (2). VISTA is a Type I transmembrane protein (3) consisting of a single N-terminal immunoglobulin (Ig) V structural domain, a stem of approximately 30 amino acids (AA), a transmembrane domain and a cytoplasmic tail containing 95 amino acids (4). The IgV structural domain of VISTA shows the highest homology with programmed cell death ligand 1 (PD-L1, a member of the B7 family) (2). The typical fold of the B7 family contains two distinct structural domains, namely, the IgV structural domain with nine β-strands and the immunoglobulin constant (IgC) structural domain with seven β-strands. In mice and humans, VISTA contains a single unusually large IgV-like structural domain (5), which has a typical disulfide bond between the putative B and F chains (3). However, as a whole, VISTA has the highest homology with programmed cell death protein 1 (PD-1, a member of the CD28 superfamily), but unlike PD-1, VISTA contains three c-terminal Src homology domain 3 (SH3) binding motifs, whereas cytotoxic T lymphocyte-associated protein-4 (CTLA-4) and CD28 contain one and two SH3-binding motifs, respectively (6). VISTA does not contain the classical immunoreceptor tyrosine-based inhibitory motif or the immunoreceptor tyrosine-based switch motif in the cytoplasmic domain. Moreover, the intracellular tail contains two potential protein kinase C-binding sites and a proline-rich motif, which may serve as a docking site (7). These cytoplasmic motifs suggest that VISTA acts as a receptor that, in a manner similar to PD-1, sends signals to VISTA-expressing cells. The similarity between VISTA and the PD-L1 IgV structural domain and the signaling potential of the VISTA receptor, P-selectin glycoprotein ligand 1 (PSGL-1) and V-set and Ig domain-containing 3 (VSIG3) suggests that VISTA may also function as a ligand (**Figure 1**) (8).

### 2.2 Expression

At the cellular level, VISTA is highly expressed in the CD11b^Hi^ myeloid cells, including granulocytes, monocytes, macrophages and dendritic cells (DCs) (5, 9, 10). Its expression is slightly lower in the lymphoid lineage, where it is expressed in γδT cells, naïve CD4+ T cells, plasma cells, CD56^low^ NK cells and forkhead box P3 (FoxP3+) CD4+ regulatory T cells (Tregs) (11). Its expression level on CD19+B cells and CD56^high^ NK cells has not yet been observed. At the tissue level, it is primarily expressed in hematopoietic tissues (i.e. spleen, thymus and bone marrow) or leukocyte infiltration-rich tissues (i.e. lungs) in mice, with a weak expression in non-hematopoietic tissues (i.e. heart, kidney, brain, muscle, testis, embryo and ovary) (12). The VISTA expression pattern is almost identical between mice and humans, with 76% homology between these two species, and is primarily restricted to hematopoietic tissues (13, 14).

### 2.3 Binding partners

As previously shown, VISTA can either act as a receptor expressed on T cells that binds to a ligand and activates the TCR-related downstream inhibitory pathways to exert an inhibitory effect on T cells or as a ligand (e.g. expressed on tumor cells) that acts in conjunction with an unknown receptor (14). Human VISTA has binding partners with proven immunosuppressive functions, such as PSGL-1, VSIG3 (15), Galectin-9 (Gal-9), V-set and immunoglobulin domain-containing 8 (VSIG8), matrix metalloproteinase 13 (MMP-13), leucine-rich repeats and immunoglobulin-like domains 1 (LRIG1) and syndecan-2.

**Figure 2:**
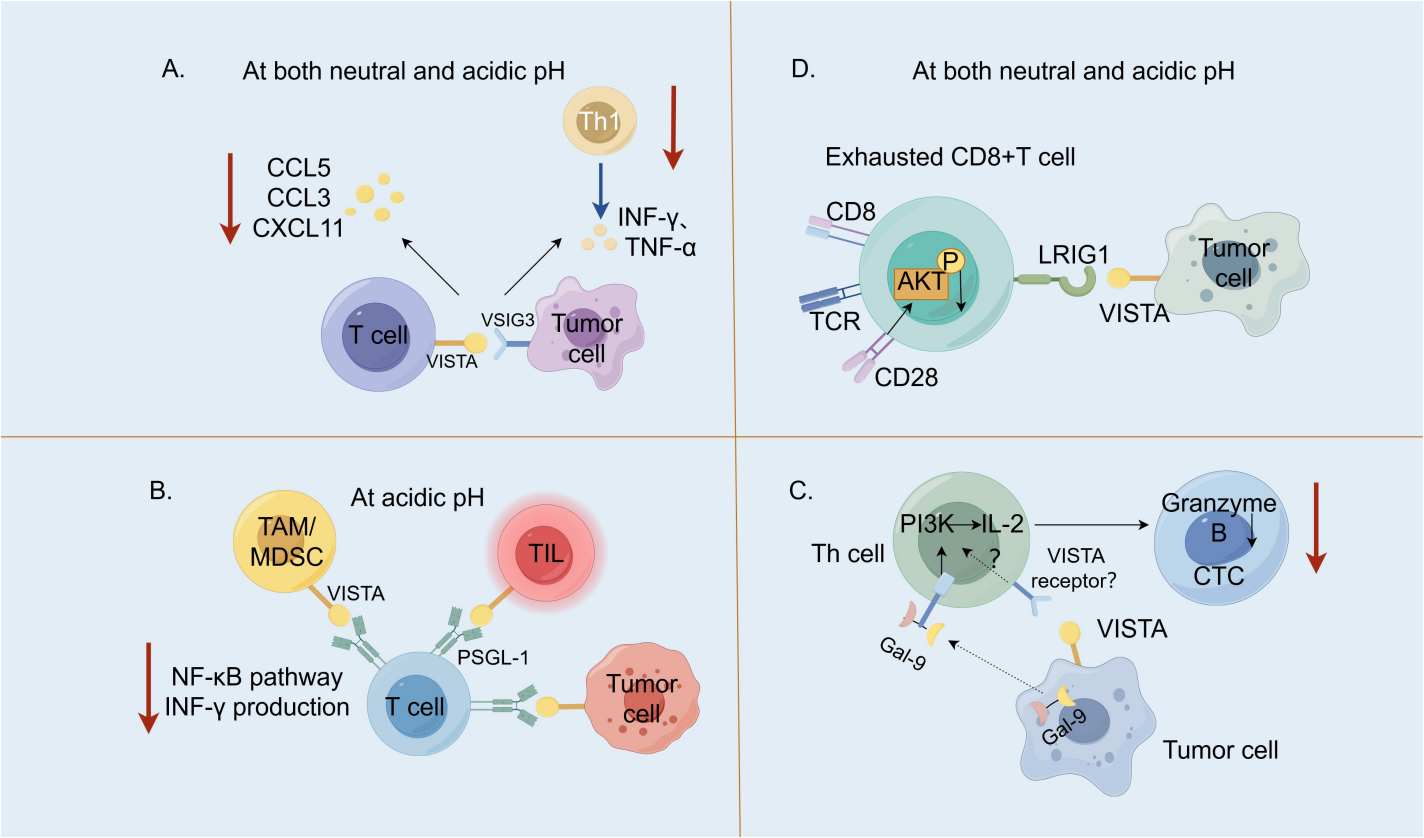
V-domain Ig suppressor of T-cell activation (VISTA) and its binding partners. **(A)**. The interaction between the V-set and Ig domain-containing 3 (VSIG3), which are expressed within the tumor cells with VISTA on the T cells, negatively regulated the secretion of CCL5/Rantes, CCL3/MIP-1α and CXCL-11/I-TAC chemokines and may inhibit the infiltration of Type 1 T helper (Th1) cells into tumor tissues (16). **(B)**. At an acidic pH, the interaction between P-selectin glycoprotein ligand 1 (PSGL-1) expressed on T cells with VISTA expressed on tumor cells, tumor-infiltrating lymphocyte (TILs) and tumor-associated macrophages (TAMs)/myeloid-derived suppressor cells (MDSCs) suppressed the T-cell activation (blocking the NF-κB signaling and reducing the IFN-γ production) and proliferation (2). **(C)**. Galectin-9 (Gal-9) produced by human cancer cells activates the PI3K and IL-2 production in the Th cells. The human cancer cells expressing both Gal-9 and VISTA suppress both the helper and cytotoxic T-cell (CTC) activities (17). **(D)**. VISTA inhibits T-cells by engaging immunoglobulin-like domain 1 (LRIG1) at both neutral and acidic pH (18). LRIG1 expressed in T cells has a broad impact on T-cell receptor (TCR) signaling and T-cell activation; when LRIG1 binds VISTA, CD28 expression is degraded and AKT activation is suppressed. CCL5 [chemokine (C–C motif ligand 5], CCL3 [chemokine (C–C motif) ligand 3], CXCL11 (C–X–C motif chemokine 11), NF-κB (nuclear factor kappa B), IFN-γ (interferon-gamma), PI3K, phosphoinositide 3-kinase and AKT, protein kinase B. (By Figdraw).

VSIG3 is a member of the immunoglobulin superfamily (IgSF), which is also known as the immunoglobulin superfamily 11 (IgSF11) and highly expressed in the brain and the testes (15). VSIG3 is a Type I transmembrane protein with an extremely low expression in normal tissues; however, its expression is significantly up-regulated in intestinal-type gastric, colorectal and hepatocellular carcinomas, suggesting that it serves as an important tumor-associated antigen. Wang et al. (16) were the first to report that VSIG3, a novel ligand for VISTA, negatively regulates the secretion of chemokine (C–C motif) ligand 5 (CCL5)/Rantes, chemokine (C–C motif) ligand 3 (CCL3)/MIP-1α and C–X–C motif chemokine 11 (CXCL-11)/I-TAC chemokines in human peripheral blood mononuclear cells and T cells. It may inhibit the infiltration of Type 1 helper T (Th) cells in tumor tissues, which is the major helper T-cell subset involved in the anti-tumor response (**Figure 2A**). The VISTA receptor knockdown on CD3+ cells using siRNA revealed that the VISTA expression on T cells correlated with the inhibitory effect of VSIG3 on the T-cell cytokine secretion. This suggests that blocking the VSIG3/VISTA pathway represents a novel cancer immunotherapy strategy. A study comparing the affinity of VISTA for VSIG3 at different pH revealed that at pH 6.0, the binding affinity of VISTA for VSIG3 decreased four-fold compared to pH 7.4 (4). Xie et al. (15) solved the crystal structure of the extracellular region of the human VSIG3 protein produced in *Escherichia coli* at a resolution of 2.64 Å, which is the first time that a high-resolution structure of VSIG3 was reported. Ghouzlani et al. (19) found that the expression of the IgSF11 gene in high-grade glioma tissues was significantly up-regulated, and was positively correlated with VISTA. There was a high infiltration of CD4 and CD8 cells, but they often showed limited effector functions, which may be related to the immunosuppressive effect of IGSF11, but this still needs to be confirmed.

PSGL-1 is a disulfide-linked homodimeric Type I transmembrane glycoprotein (14) encoded by the SELPG gene, whose expression primarily occurs on hematopoietic cells. Human PSGL-1 is highly expressed on almost all leukocytes, with a lower expression on B cells (20). Tinico (21) et al. reported that PSGL-1 is involved in inhibiting TCR activation, reducing the interleukin (IL)-2 production and up-regulating other co-suppressors, such as PD-1, in a mouse model of chronically infected lymphocytic choroid plexus meningitis virus, which is evidence that PSGL-1 is an immune checkpoint receptor. Under acidic conditions, VISTA histidines are protonated, facilitating ionic interactions with negatively charged glutamic acid residues and sulphated tyrosine residues in PSGL-1. At pH 7.4, the histidine side chain of VISTA is unphosphorylated and does not bind to PSGL-1. It was hypothesized that the PSGL-1/VISTA pathway may be important for inhibiting T-cell activation under acidic conditions (2). An acidic pH-selective VISTA mAb(BMS-767) (22) that blocked the PSGL-1/VISTA interaction increased the interferon-gamma (IFN-γ) production, nuclear factor kappa B (NF-κB) phosphorylation and cell proliferation in the human CD4+ T cells cultured *in vitro* with VISTA-expressing cells (**Figure 2B**).

Galectin-9 (Gal-9) is a member of the Gals family (23), which is structurally characterized by a carbohydrate recognition domain that specifically binds to polysaccharides containing β-galactosides and exerts both intracellular and extracellular effects (24). Gal-9 was first identified as a potent eosinophil chemotactic factor widely distributed in the liver, small intestines, lungs, spleen and other organs. There are three natural isoforms of human Gal-9, namely, Gal-9 (S), Gal-9 (M) and Gal-9(L). The differences between the Gal-9 isoforms are related to the linker, which primarily affects the ability of the isoforms to bind to glucose ligands (25). It does not affect the function of Gal-9 in recruiting eosinophils and inducing apoptosis in T cells. Yasinska et al. (26) biophysically demonstrated the interaction between Gal-9 and VISTA and through immunoprecipitation experiments found a high-affinity interaction between the Gal-9 ligand and VISTA. Soluble VISTA significantly enhances the pro-apoptotic effects of soluble galectin-9 in T cells. This occurs due to changes in cell polarization/membrane potential, which may attenuate the capability of T cells to release granzyme B from the cell. By coculturing LN18 high grade glioblastoma cells and Jurkat T cells, Schlichtner et al. (17) reported that neutralization of VISTA led to upregulation of phosphoinositide 3-kinase (PI3K) activity and IL-2 secretion in Jurkat T cells and neutralization of either galectin-9 or VISTA led to decreased viability of LN18 cells as well as increased granzyme B release. They demonstrated that VISTA enhanced the immunosuppressive effects of Gal-9 by attenuating the PI3K activity/IL-2 production, thereby enabling Gal-9 to suppress the activity of the Th and cytotoxic T cells (CTLs) (**Figure 2C**). Gal-9 plays an important role in NK cell activation and release of IFN. The differences in the effector functions of Gal-9+ natural killer (NK) cells between mice and humans should be considered under various physiological and pathological conditions. A recent study (27) revealed the expansion of Gal-9+ NK cells in the tumor tissue of melanoma mice and found that the presence of Gal-9 was associated with enhanced expression of the cytotoxic effector molecules granzyme B and perforin. In a separate article (28), when 10% human AB serum (ABS) is utilized as a culture supplement, the application of recombinant Gal-9 has been shown to induce the expression of Tim-3 on NK-92MI cells. Additionally, an elevated expression of Tim-3, CD69, and natural killer cell group 2D (NKG2D)-activating receptors suggests a dose-dependent activation of these cells. When considering Gal-9 as a target for cancer immunotherapy, it is imperative to meticulously characterize the modulatory impacts of Gal-9 on the effector cells involved in the anti-tumor response.

LRIG1 is a transmembrane protein that negatively regulates the epidermal growth factor receptor signaling pathway. Unlike PSGL-1, LRIG1 binds to VISTA at an acidic and neutral pH (18). It occurs in the same cell (cis) and in different cells (trans), and VISTA may be involved in the inhibitory signaling exerted by LRIG1 to drive the quiescence of tumor-responsive CTLs. Anti-LRIG1 monoclonal antibodies disrupt the interaction between VISTA and LRIG1, resulting in an increased proliferation of immune cells, increased polarization of M1-type macrophages, and an increase in pro-inflammatory cytokines, particularly IFN-γ, which promotes anti-tumor effects (**Figure 2D**) (29).

In addition to this, WANG (16) et al. concluded by an Enzyme-linked Immuno Sorbent Assay (ELISA) binding screening assay that VISTA was not related to the VISG family except for VSIG3. However, Molloy (30) et al. first reported the discovery of an interaction between VISTA and VSIG8,and suggested that agonism or antagonism of VSIG8 could be used for the treatment of cancer, autoimmunity, metabolic or inflammatory diseases. Then Chen (31) reported that VSIG8 interacts with VISTA and, using experimental methods such as ELISA, Microscale Thermophoresis (MST) and coimmunoprecipitation (Co-IP), also inhibits T cell function. Fu et al. (32) found that the programmed death-1 homologue, PD-1H (namely VISTA), is an MMP-13 receptor in osteoblasts. Silencing PD-1H or using PD-1H−/− bone marrow cells attenuated the MMP-13-enhanced osteoclast fusion and bone resorption activity. MMP-13 is overexpressed in various tumors, including multiple myeloma, breast, lung, gastric and colorectal cancers and is associated with poor prognosis, lymph node metastasis and shorter overall survival in cancer patients (33).

## 3 Immunoregulatory mechanisms

### 3.1 General immune responses

**Figure 3:**
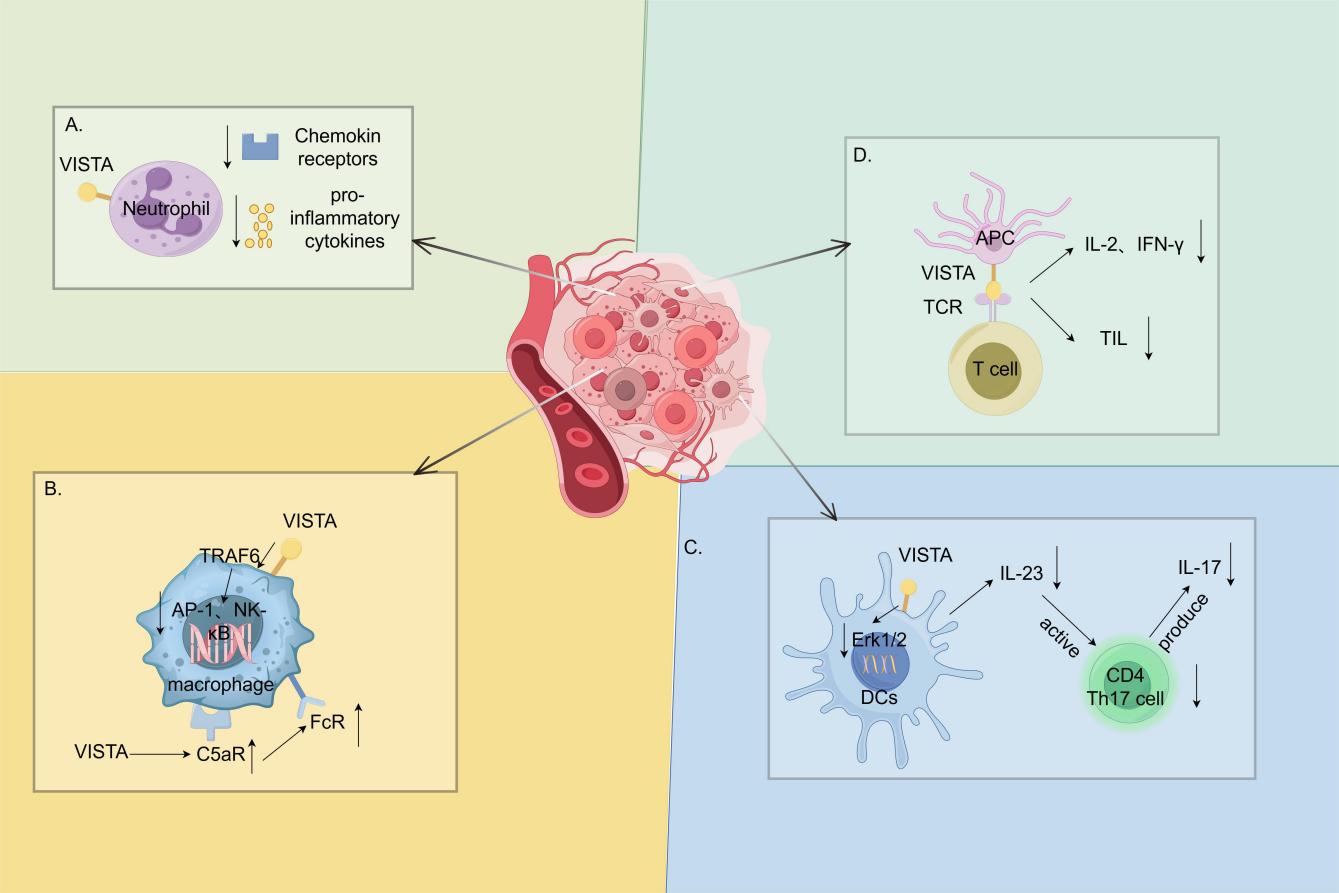
Immune function of the V-domain Ig suppressor of T-cell activation (VISTA) highly expressed in myeloid cells. **(A)**. The V-domain Ig suppressor of T-cell activation (VISTA) in neutrophils suppresses inflammation by decreasing the chemokine receptor and the pro-inflammatory cytokine expression (34). **(B)**. VISTA has a dual role in macrophages. On the one hand, it inhibits the release of pro-inflammatory cytokines through the MAPKs/AP-1 and IKKα/β/NF-κB signaling pathways (11). On the other hand, it ensures the expression of C5a receptors on the macrophage surface in mice, which forms immune complexes and promotes inflammatory responses (35). **(C)**. VISTA, which has a regulatory role in the IL-23/IL-17 axis, regulates the IL-23 production in dendritic cells (DCs) by attenuating the ErK1/2 activation. It subsequently inhibits the CD4 T helper lymphocyte 17 (Th17) activation and the IL-17 production (36). **(D)**. Interactions with APCs and T cells inhibit antigen-specific T-cell activation (5) and reduce the IL-2 and IFN-γ production, as well as the number of tumor-infiltrating CD8+ T cells (37). MAPKs, mitogen-activated protein kinases; AP-1, activator protein 1; IKK, IKB kinase complex; NF-κB, nuclear factor kappa B. (By Figdraw).

VISTA is highly expressed in the myeloid lineage and regulates various myeloid cell functions. In neutrophils, VISTA suppresses inflammation by inhibiting chemotaxis and the release of pro-inflammatory cytokines IL-6, TNF-α and monocyte chemoattractant protein-1 (MCP-1) (**Figure 3A**) (34). It is highly expressed in macrophages, which play a dual role in the inflammatory response. Although VISTA inhibits the pro-inflammatory cytokines released through the mitogen-activated protein kinases (MAPKs)/activator protein 1(AP-1) and the IKB kinase complex (IKK) α/β/NF-κB signaling pathways (11), it ensures the expression of the C5a receptors on the macrophage surface in mice, thereby resulting in the formation of immune complexes and promoting an inflammatory response (35). It also upregulates the expression of the C–C chemokine receptor Type 2 (CCR2) to promote an inflammatory response (**Figure 3B**) (38). Li et al. identified an important regulatory role for VISTA in the IL-23/IL-17 axis. VISTA regulates the IL-23 production in DCs by regulating the ErK1/2 activation and negatively regulating the IL-7-mediated homeostasis of the CD27-γδT cells, as well as the γδT cell responses to the TCR- or IL-23/IL-1β-mediated stimuli (**Figure 3C**). These effects collectively result in excessive psoriasis-like inflammation in Vsir−/− mice (36).

VISTA is expressed on lymphocytes at a lower level compared with myeloid cells and acts as an immune checkpoint that inhibits T-cell activation and proliferation, with significant inhibitory effects on both resting and activated CD4+ and CD8+ T cells. As a ligand, it interacts with APCs and T cells to inhibit antigen-specific T-cell activation (5) and reduce IL-2 and IFN-γ production and the number of tumor-infiltrating CD8+ T cells (37). VISTA also promotes the conversion of naïve T cells to Foxp3 Treg T cells, thereby acting as an immunomodulator (**Figure 3D**) (13).

VISTA−/CD4+ T cells increase T-cell proliferation and the production of IFN-γ, tumor necrosis factor α (TNF-α) and IL-17A compared with untreated controls, suggesting that it is a suppressor receptor for the CD4+ T cells (39). Furthermore, when naïve murine CD4+ T cells were exposed to foreign antigens and differentiated into memory CD4+ T cells, the VISTA expression on the CD4+ T cells was decreased (8). Compared with the wild-type (WT) mice, the Vsir−/− mice exhibited impaired activation-induced cell death and resulted in fewer peripheral T-cell deficits and the emergence of an autoimmune phenotype (34).

### 3.2 Mechanisms of VISTA in tumor immunity

ICRs are usually highly expressed in tumor cells. Among the tumor types in The Cancer Genome Atlas (TCGA) (40), the highest expression of human VISTA was observed in epithelioid mesotheliomas, including both tumor and inflammatory cells (39) and in human lung, kidney, ovarian, endometrial and colorectal cancers, as well as other diseases (37, 41–43). However, Mercier et al. (44) reported that in the tumor microenvironment, VISTA is highly expressed in the myeloid and Foxp3+ CD4+ regulatory cells, but not in the tumor cells.

VISTA was highly expressed in the most hypoxic regions of the mouse colon CT26 tumors. Hypoxia is a mediator of the tumor immune escape and treatment resistance. Deng et al. (45) found that hypoxia upregulates VISTA on myeloid-derived suppressor cells (MDSCs) through hypoxic inducible factor 1α (HIF-1α) binding to conserved hypoxia-responsive elements in the VISTA promoter. This inhibits the T-cell activity and facilitates the completion of immune escape by the tumor cells. MDSCs are a heterogeneous cell population (46). Wang et al. (47) found that VISTA is highly expressed in MDSCs, and the VISTA knockdown significantly attenuates the MDSC-mediated inhibition of T-cell proliferation. This suggests that the upregulation of VISTA may be an alternative mechanism for the immunosuppressive activity of MDSCs. In the tumor microenvironment, the presence of M2 tumor-associated macrophages (M2-TAM) is associated with poor clinical prognosis, resistance to therapy (48, 49), and poor antigen presentation (50). Lin et al. (51) found that the ectopic expression of VISTA drives the phenotypic shift of monocytes to M2 macrophages, down-regulates the signal regulatory protein alpha (SIRPα), reduces the IL-1β levels and increases the anti-inflammatory cytokine IL-10 levels, thereby resulting in an immunosuppressive microenvironment and promoting tumor progression *in vitro* (52).

VISTA showed surprising results when combined with other therapies. The large CT26 tumors showed complete adaptive resistance to anti-PD-1/CTLA-4 in combination therapy (53), but the addition of anti-VISTA resulted in the rejection of half of the tumors. Therefore, VISTA may serve as a novel target for circumventing immune checkpoint inhibitor (ICI) resistance. With respect to the ICI resistance, VISTA differed from CTLA-4 and PD-1 in that anti-VISTA treatment promotes co-stimulatory factors and decreases T-cell resting regulators. Zhang et al. (54) found that VISTA is expressed in tumor-associated neutrophil (TAN) cells and significantly increases in TAN cells during radiotherapy (RT). The combination of anti-VISTA and RT synergistically inhibited tumor growth and significantly reduced the elevated aggregation of TANs, M-MDSCs and M2-TAMs following RT. The combination group also enhanced the infiltration and activation of CD8+ tumor-infiltrating lymphocytes (TILs). Toll-like receptor 3 (TLR3), a TLR family member, mediated the transcriptional induction of pro-inflammatory cytokines and chemokines. A VISTA-specific monoclonal antibody (13F3) specifically enhanced the ability of a TLR3 agonist adjuvant to induce macrophage activation *in vitro*. In a mouse model of bladder cancer, the 13F3 and TLR3 combination reduced the frequency of the anti-inflammatory macrophages within the tumor and the immunosuppressive transforming growth factor-β1 (TGF-β1) while increasing the CD8+T/Treg ratio (55) exhibiting a high clinical translational potential.

## 4 Research progress in hematologic diseases

Hematologic diseases show similarities and differences from solid tumors in terms of pathogenic mechanisms and therapeutic approaches. Tumor immunotherapy has become an active research area in recent years and the emergence of ICIs has become a major focus in tumor immunotherapy. Clinical evidence has demonstrated that biologicals, such as PD-1 monoclonal antibodies, produce remarkable therapeutic effects and VISTA, as an immune checkpoint receptor for which no drug has yet been developed, has not been examined in hematologic tumors.

### 4.1 Acute myeloid leukemia

Acute myeloid leukemia (AML) is a heterogeneous disease caused by the abnormal proliferation of clonal hematopoietic cells. Previous studies revealed that the overall survival (OS) rate of AML patients at 5 years is approximately 30%. After the definitive diagnosis of AML, the primary treatment goal is to achieve a complete response, which reduces the leukemic load. This is followed by post-remission consolidation therapy, which can either be chemotherapy or hematopoietic stem cell transplantation. Abnormal immune microenvironment is an important part of the pathogenesis of AML. In recent years, the efficacy of AML has improved significantly, but the results are not satisfactory. Immunotherapy is emerging as a combined treatment approach with classic intensive chemotherapy regimens. In NCT04353479, a PD-1 inhibitor was used in conjunction with decitabine to treat elderly patients with relapsed and refractory AML. In NCT03066648, TIM-3 monoclonal antibody MBG 453 is being explored for its safety and tolerability as a monotherapy or in combination therapy among patients with AML and intermediate or high-risk myelodysplastic syndromes (MDS).

VISTA is highly expressed in AML. Pagliuca (56) reported a linear increase in the VISTA expression throughout myeloid differentiation by analyzing multiple transcriptional datasets. A high enrichment was observed in the granulomatous mononuclear and mononucleated differentiated AML. The VISTA expression was increased in both leukemic and T cells in relapsed cases within 2 years of diagnosis compared to patients in long-term remission (>5 years after the standard chemotherapy regimen). The upregulation of VISTA on leukemic and T cells may contribute to the weakening of the immune surveillance mechanism against AML cells. A statistically significant increase in the MDSCs was observed in AML patients compared to healthy controls (57, 58). VISTA was highly expressed in the MDSCs of AML patients and the siRNA-mediated VISTA knockdown significantly reduced the MDSC-mediated suppression of the CD8 T-cell activity in AML (47). The MDSC expression of VISTA was strongly and positively correlated with the T-cell expression of PD-1, but the underlying mechanism is unclear.

VISTA may play a role in the immune escape of AML. Kim et al. (59) established a myeloid leukemia cell line in mice. Compared with WT mice that did not express VISTA, mice transduced with lentiviral plasmids expressing VISTA had faster-growing tumors. However, no significant growth differences were observed in immunodeficient mice. Mice with myeloid leukemia were treated with the specific VISTA mAb 13F3 and a control mAb. The 13F3-treated mice showed a markedly slower tumor growth, whereas the anti-leukemic effect of 13F3 in WT B6 mice was inhibited by removing T cells with CD4 and CD8 monoclonal antibodies. The NK-cell clearance had no effect. Flow cytometry revealed a significant increase in the percentage of granzyme B+CD8+T cells as well as effector memory phenotype (CD44+CD62L−) CD8+ T cells, without increase in infiltrating CD4+, CD8+ immune cells. The results suggest that VISTA inhibition improves the quality of the T-cell response instead of increasing T-cell infiltration in this model.

The signal transducer and activator of transcription 3 (STAT3) is a member of the B7 family that may be associated with the VISTA expression (60). Mo et al. (61) identified two distinct binding peaks for STAT3 in the promoter and the first intron of the VISTA gene using the cis-anti group DataBrowser database. They found an association between STAT3 and VISTA binding. Blocking VISTA reduced the STAT3 activation, decreased the STAT3-dependent peptide synthesis, and disrupted the mitochondrial respiration and MDSC amplification (62). This suggests that it may be possible to play an immunotherapeutic role in AML by inhibiting VISTA, and perhaps a combination of STAT3 and VISTA inhibitors could obtain better therapeutic results.

### 4.2 Multiple myeloma

Multiple myeloma (MM) is the second most common hematologic malignancy characterised by the abnormal proliferation of clonal plasma cells in the bone marrow. Clinical manifestations primarily include anemia, hypercalcemia, bone disease and renal impairment (63). Currently, MM is treated with induction therapy, and early sequential autologous stem cell transplantation is recommended after effective induction therapy; otherwise, treatment is continued into the maintenance phase after consolidation therapy. Despite the emergence of proteasome inhibitors and immunomodulators in recent years, which prolong the survival of MM patients, it remains incurable.

Huang et al. (64) demonstrated that VISTA is closely associated with the induction and development of exhausted T cells in MM. They examined the VISTA expression on different T-cell subsets and observed a high expression along with other immune checkpoints in the peripheral blood (PB) and bone marrow (BM) of MM patients. The VISTA+ T and VISTA+, TIM3+, TIGIT+ PD-1+ T cells were highly expressed in the PB compared with that in the BM of MM patients. This is in contrast to previous hypotheses that the BM exerts a greater inhibitory effect on T cells. However, the TIM3+, TIGIT+ and PD-1+ T cells alone were higher in the BM, suggesting that VISTA has a more pronounced T-cell depleting effect in the PB of MM patients, although the exact mechanism of its upregulation is unclear. Through clinical data statistics and biochemical characterization, they concluded that the increased VISTA expression is associated with poor clinical outcomes. Mutsaers et al. (65) found that the VISTA expression is associated with poor OS in MM patients. Through immunofluorescence images, they concluded that the major source of VISTA was CD11b+ cells in MM patients. By contrast, the VISTA expression was not observed in the T cells within the tumor.

Amyloid light-chain (AL) amyloidosis is a rare plasma cell disease that belongs to the group of monoclonal immunoglobulin disorders. It is characterized by the proliferation of clonal plasma cells and the production of monoclonal immunoglobulins and often results in the dysfunction of vital organs, such as the heart and the kidneys (66). Patients with AL amyloidosis had a significantly higher percentage of VISTA+ T cells in their PBs compared to healthy controls, suggesting that it may be a potential target for the reversal of AL amyloidosis and restoring exhausted T cells in patients (67).

### 4.3 Lymphoma

Lymphoma is a group of malignant tumors originating from the lymph nodes or other lymphoid tissues. These tumors may be divided into two major categories: Hodgkin’s lymphoma and non-Hodgkin’s lymphoma. Histology reveals the neoplastic proliferation of lymphocytes and/or histiocytes. The clinical presentation is typical of a painless lymph node enlargement. The cellular morphology of lymphoma is extremely complex as 80 subtypes are recognized in the 2008 World Health Organization’s New Classification of Lymphoma. The clinical manifestations are inconsistent, and the treatment regimens vary due to the different sites and ranges of lesions.

Studies have identified the expression of VISTA in several lymphomas. For example, peripheral T-cell lymphomas (PTCL) (68) account for 10%–15% of non-Hodgkin’s lymphomas and are characterized by high aggressiveness and a poor prognosis. The VISTA expression was not observed in lymphocytes from benign primary or secondary germinal centers of PTCL and was rare in the tumor microenvironment. The authors suggested that this may be related to the general absence of p53 in PTCL; however, it cannot be ruled out whether immunohistochemistry was insufficient for detecting low expression levels. The extra-nodal natural killer/T-cell lymphoma (ENKTCL) is a rare, but aggressive subtype of PTCL derived from NK or γδT cells (69) He et al. (70) found high VISTA expression associated with distal lymph node (LN) metastasis, advanced Ann Arbor stage, high nomogram-revised index and a high prognostic index of NK/T cells. Primary nasal tumors had a higher VISTA expression compared to other primary tumors. In addition, a significant correlation existed between the PD-L1 and VISTA expressions, with VISTA being synergistic with PD-L1, which could be a poor prognostic indicator for ENKTCL. The VISTA expression in other lymphoma types awaits further exploration (**Table 1**).

**Table 1:** Expression of VISTA in several hematological diseases.

Disease type	Pattern of the VISTA expression	Citation
AML (47, 56)	Upregulation in AML compared with healthy controls (p = 0.0002), particularly in morphological subtypes with myelomonocytic and monocytic differentiations	Pagliuca (2022)
	Expression in MDSCs of the peripheral blood, 54.3% AML *vs* 33.3% in healthy controls (p = 0.0262)	Wang (2018)
MM (64, 65)	Increased percentage of VISTA+CD3+ (p < 0.001). VISTA+CD4+ (p < 0.001) and VISTA+CD8+ (p < 0.001) T-cell in MM compared with HIs	Huang (2022)
	Expression correlates with OS (p = 0.005) and predominantly on CD11b+ myeloid cells	Mutsaers (2021)
AL amyloidosis (66)	High expression in CD3+, CD4+ and CD8+ T cells and Tregs in PB of AL amyloidosis patients	Wang (2024)
PTCL (68, 70)	Expression in 5% of PTCL-NOS cases (n = 37), 66% of cases of EATL (n = 3) and 33% of AITL cases (n = 6)	Murga-Zamalloa (2020)
	High expression (≥27.5%) is significantly correlated with distal LN metastasis (p = 0.004) in ENKTCL	He (2021)

VISTA, V-domain Ig suppressor of T-cell activation; AML, acute myeloid leukemia; MDSC, myeloid-derived suppressor cells; MM, multiple myeloma; HIs, healthy individuals; OS, overall survival; AL, amyloid light-chain; PB, peripheral blood; PTCL, peripheral T-cell lymphomas, PTCL-NOS, peripheral T-cell lymphoma, not otherwise specified; EATL, enteropathy-associated T-cell lymphoma; AITL, angioimmunoblastic T-cell lymphoma; ENKTCL, extra-nodal natural killer T-cell lymphoma; LN, lymph node.

## 5 Current status of VISTA drug research

Drugs targeting VISTA are currently in the preclinical stage and include oral small-molecule drugs and VISTA monoclonal antibodies. CA-170 is an oral small-molecule dual antagonist that selectively targets PD-L1 and VISTA. It induces the proliferation of IFN-γ in T cells specifically inhibited by PD-L1 and VISTA (71). Preclinical data show that CA-170 exhibits antitumor effects similar to PD-1 or VISTA antibodies in various tumor models. Toxicological studies have demonstrated its safety. Results from the phase I dose-escalation study (NCT02812875) revealed that patients diagnosed with non-small cell lung cancer, head and neck cancer, or Hodgkin’s lymphoma were randomized to receive either 400 mg or 800 mg of CA-170. An outstanding clinical benefit rate (CBR) and progression-free survival (PFS) were observed at the 400 mg dose, but the results were not officially announced after the trial ended in 2020.

Several other monoclonal antibodies are also undergoing clinical trials, primarily for solid tumors, either alone or in combination with PD-1 monoclonal antibodies. SNS-101 is a highly selective monoclonal IgG1 antibody. Preclinical data suggest that it inhibits interaction with PSGL-1 (72). HMBD-002 is the first Fc-independent IgG4-type anti-VISTA antibody developed by Hummingbird Bioscience. Developed under the guidance of AI, this antibody targets a specific conserved epitope on the C-C’ loop unique to VISTA. It has demonstrated potent inhibition of tumor growth in preclinical humanized mouse models of colorectal, lung, and breast cancers. Hummingbird has initiated a multicenter phase 1/2 clinical trial (NCT05082610) enrolling patients with malignant solid tumors. The primary objective of the phase I clinical trial was to determine the recommended phase II dose (RP2D) of HMBD-002 as a single agent and in combination with the anti-PD-1 monoclonal antibody pembrolizumab in patients with advanced solid malignancies. A phase II clinical program will evaluate HMBD-002 alone or in combination with anti-PD-1 antibody in patients with triple-negative breast cancer, non-small cell lung cancer, and other malignancies known to express VISTA (73). KVA12123, Kineta’s immuno-oncology drug targeting VISTA, cleared the first three monotherapy dose levels and was well tolerated, with no dose-limiting toxicities (DLT) or cytokine-related adverse events observed. Additionally, KVA12123 exhibited a greater-than-dose-proportional pharmacokinetic profile, achieving more than 90% VISTA receptor occupancy (RO) in patients within the 30 mg dosing cohort.

CI8993 (formerly known as JNJ-61610588) is an anti-VISTA monoclonal IgG1κ antibody with an active IgG1 Fc domain. Preclinical studies have demonstrated that CI-8993 increases the number of peripheral tumor-specific T cells and enhances the infiltration, proliferation, and effector functions of tumor-reactive T cells in the TME. In the phase I clinical trials in 2016, one patient developed transient dose-limiting side effects associated with cytokine release syndrome (CRS). Curis later announced results from the phase I monotherapy study (NCT04475523) of CI-8993 in relapsed or refractory solid tumors. In this study, 13 patients demonstrated a favorable safety profile, with no dose-limiting toxicities observed in the 0.15 mg/kg and 0.3 mg/kg dose groups (i.e., the dose levels at which CRS was present in 2016). Curis is enrolling patients with metastatic or unresectable, relapsed and/or refractory malignant solid tumors (non-lymphoma) to determine the maximum tolerated dose (MTD) of full-dose CI-8993 and to explore the pharmacokinetic/pharmacodynamic relationship at higher doses (74).

## 6 Discussion

As a novel immune checkpoint receptor, VISTA exhibits unique expression pattern and mechanism of action. For example, while PD-1 and Tim-3 are highly expressed in immune cells, VISTA is expressed in the myeloid lineage and usually has a co-expression relationship with other immune checkpoint receptors. This suggests that VISTA may serve as a target for overcoming drug resistance after immunotherapy. Whether it is effective in combination with drugs, such as PD-1 monoclonal antibody, remains to be determined. VISTA plays a role in immunosuppression and immune quiescence, which has potential for treatment of tumors and autoimmune diseases. Nonetheless, the pathogenic mechanism of VISTA in different diseases remains unclear, which should be the focus of future studies. In addition, the VISTA ligands, VSIG3 and PSGL-1, are associated with acidity, whether the two ligands have competing roles *in vivo*, who is dominant in different cell types and environments. Downstream pathways signaling pathways, and other mechanisms will help to establish a more specific and systematic screening approach to improve the success rate of drug discovery and development.
